# Psychiatric disorders in adolescents and young adults with Down syndrome and other intellectual disabilities

**DOI:** 10.1186/s11689-015-9101-1

**Published:** 2015-03-01

**Authors:** Elisabeth M Dykens, Bhavik Shah, Bruce Davis, Courtney Baker, Taylor Fife, Jeri Fitzpatrick

**Affiliations:** Vanderbilt Kennedy Center for Research on Human Development, Vanderbilt University, 1 Magnolia Circle, Peabody Box 40, Nashville, USA; Departments of Psychology and Human Development, Psychiatry, and Pediatrics, Vanderbilt University, 1 Magnolia Circle, Peabody Box 40, Nashville, USA; Department of Psychiatry, UCLA David Geffen School of Medicine, Los Angeles, USA

**Keywords:** Down syndrome, Intellectual disabilities, Depression, Psychosis, Catatonia

## Abstract

**Background:**

Relative to other aspects of Down syndrome, remarkably little is known about the psychiatric problems experienced by youth and young adults with this syndrome and if these problems differ from others with intellectual disabilities. Yet adolescence and young adulthood are particularly vulnerable time periods, as they involve multiple life transitions in educational, medical, and other service systems.

**Methods:**

This study compared the psychiatric diagnoses of 49 adolescent and young adult patients with Down syndrome to 70 patients with other intellectual disabilities (IDs). The groups were similar in age, gender, and level of intellectual impairment. The 119 participants, aged 13 to 29 years (*M* = 21) were evaluated in one of two specialized psychiatric clinics.

**Results:**

In contrast to previous literature, those with Down syndrome versus other IDs had significantly higher rates of psychosis NOS or depression with psychotic features (43% versus 13%). Unlike the ID group, psychosis was predominantly seen in females with Down syndrome. Marked motoric slowing in performing routine daily activities or in expressive language was manifested in 17% of patients with Down syndrome. No group differences were found in anxiety or depressive disorders, and the ID group had significantly higher rates of bipolar and impulse control disorders.

**Conclusions:**

These preliminary observations warrant further studies on genetic, neurological, and psychosocial factors that place some young people with Down syndrome or other IDs at high risk for severe psychiatric illness.

## Background

A growing literature describes the behavioral phenotypes of people with neurodevelopmental disorders, but not all neurodevelopmental disorders receive equal research attention. Down syndrome, for example, is understudied both in relation to rare, clinically severe disorders and to more prevalent conditions such as autism spectrum disorders, or attention deficit hyperactivity disorder [1]. Not surprisingly then, research gaps are increasingly identified in the Down syndrome literature [2,3].

One salient research gap concerns co-occurring mental health disorders in Down syndrome and how these are manifested in different age groups [2]. Older adults with Down syndrome, for example, are relatively well studied because of their increased risks of Alzheimer’s disease, attributed to the third copy and overexpression of the amyloid precursor protein gene on chromosome 21 (for a review, see Zigman [4]). Depressive symptoms in this older age group have also been described, and careful evaluations are necessary to differentiate a depressive disorder from the early clinical signs of dementia [5]. On the other end of the developmental spectrum, young children with Down syndrome appear to have lower overall rates of disruptive behaviors as well as distinctive social, motivational, and attention profiles [6-8]. In contrast to older adults or children, remarkably little is known about the behavioral or emotional disorders of adolescents or young adults with Down syndrome [2,3].

This research gap is an important one to fill as young adults with intellectual disabilities, including Down syndrome, are transitioning out of educational settings and entering adult service systems that are often more fragmented and less readily available [9]. These young adults also leave pediatric care, and problems transitioning to adult medicine are associated with persistent health care disparities in the intellectual disability population [10]. People with intellectual disability (ID) and psychiatric problems may be more sensitive or negatively impacted by life events [11], further justifying the need for studies in the adolescent to young adult transitional period.

This study identified psychiatric diagnoses in 13- to 29-year-old adolescent and young adult patients with Down syndrome and other intellectual disabilities, but with an eye toward also addressing two inconsistencies in the Down syndrome literature. First, consistent with higher risks of psychopathology in the ID versus general population, early studies found that adults with Down syndrome were especially prone to depressive or anxiety disorders [12-15]. In contrast, recent reports suggest that while some adolescents or young adults may become more withdrawn or quiet over time [16,17], rates of depressive disorders are no higher in Down syndrome than others with intellectual disabilities [18].

A second and related research discrepancy relates to the severity of problems as reflected in community versus clinic samples. Relative to others with intellectual disabilities, much lower rates of behavior or emotional problems are consistently found in non-referred, community samples of individuals with Down syndrome [5,18,19]. Such studies find that impulsivity, aggressive, and disruptive behaviors are particularly low in Down syndrome and lessen over time [16,19,20].

Studies of clinic or hospitalized patients with Down syndrome paint a different picture involving more severe or unusual psychiatric concerns. Prasher [21], for example, identified ‘young adult disintegrative syndrome’ characterized by depression, withdrawal, and significant regression in cognitive, language, or motor functioning in an unspecified number of young patients with Down syndrome. Similarly, Devenny and Mathews [22] conducted a medical chart review of 197 patients with Down syndrome and identified 14 adolescents with regressions in cognitive and adaptive skills, as well as with depression, aggression, compulsivity, and withdrawal. Finally, Charlot *et al.* [23] identified what they called ‘obsessional slowness’ in performing routine daily living skills in 11 young adult patients with Down syndrome. Motoric freezing and tics were also observed in these patients, who did not appear to lose skills but to perform them at a markedly slower pace, features strongly suggestive of catatonia.

These clinic observations warrant further study as they fall outside the scope of the usual Down syndrome phenotype and may provide new insights into the range of phenotypic expression in this syndrome. This preliminary study compares the types and correlates of psychiatric disorders in adolescent and young adult clinic patients with Down syndrome versus intellectual disabilities.

## Methods

### Participants

This study enrolled 119 adolescent or young adult patients with intellectual disabilities (65 males, 54 females) aged 13 to 29 years (*M* = 21.73 years; SD = 4.27) who were seen in one of two specialized psychiatric clinics. Of these, 49 had documented etiologies of Down syndrome, and 70 had other IDs. Most members of the ID group, 78%, had unknown or undocumented causes, while 15% had histories of birth trauma, prenatal exposures, and neural injuries, and 7% had genetic syndromes (one case each of Lowe, Smith-Magenis, fragile X, 22q deletion, and Cornelia de Lange syndromes).

Because co-occurring autism spectrum disorder (ASD) is only seen in 5% to 6% of individuals with Down syndrome [24,25], patients with primary diagnoses of ASD were not included in the other intellectual disability group. This exclusion ensured that the ID comparison group was similar to the majority of those with Down syndrome. Three additional patients with Down syndrome and ASD were also excluded from the study, resulting in 49 patients with Down syndrome and 70 with ID who did *not* have ASD.

IQ test scores were variably obtained through a brief IQ screener, the Kaufman Brief Intelligence Test-2 (KBIT-2) [26], administered during the initial clinic visit for 35 patients, or a review of patient’s records that included previous IQ scores. Given the variability in the type and timing of IQ tests, broader IQ levels were used in data analyses (see Table 1).

**Table 1:** **Characteristics of patients with Down syndrome versus other intellectual disabilities**

	**Down syndrome**	**Intellectual disabilities**	***t*** **or** ***X*** ^**2**^
*N*	49	70	
Mean age, years	21.39 (5.40)	22.07 (3.59)	*t* (123) = −.78
13 to 19 years	38%	35%	NS
20 to 29 years	62%	65%	
Gender	49% M, 51% F	58% M, 42% F	NS
Level of ID			NS
Mild	69%	73%	
Moderate	19%	15%	
Severe	12%	12%	
Race			*X* ^2^ (3) = 8.65^**^
Caucasian	69%	71%	
African American	13%	24%	
Hispanic	14%	3%	
Asian/Other	4%	2%	

^**^ *p* < .01.

As shown in Table 1, the Down syndrome versus other ID groups did not differ in age, gender, or level of intellectual impairment. The Down syndrome group had equal numbers of males and females, while males were slightly but not significantly overrepresented in the ID group. The sample was predominantly Caucasian, although significant differences were seen in the distribution of African American and Hispanic patients across groups. As such, race was examined in relation to psychiatric disorders.

### Procedures and measures

#### Clinics

Participants and their families were seen in one of two University-based, outpatient psychiatric clinics on the West coast (*n* = 40) or Mid-South (*n* = 82). Both interdisciplinary clinics specialized in the psychiatric and behavioral health needs of persons with ID, including Down syndrome. Individuals who were inappropriate clinic referrals and/or did not merit a psychiatric evaluation were not included in the study. Although both clinics served a wide age range, this study only included youth and young adults. Participants at the two clinic sites did not differ in age, gender, etiology, or psychiatric diagnoses, and as such, clinic location was not taken into account in data analyses.

#### Consent

Each clinic adhered to their respective university IRB procedures, which complied with the Helsinki Declaration. Parents or guardians provided written, informed consent for specific de-identified information derived from their clinic intake visits to be used for research purposes, and they were assured that their participation in the study was not related to ongoing clinical care. Consents and data were obtained as patients were seen over an approximate 2-year period.

#### Clinic team members

Patients were evaluated at each site by an interdisciplinary team consisting of child psychiatrists, clinical psychologists, applied behavior analysts, nurses or nurse practitioners, and social workers. Trainees in each of these disciplines rotated through the clinics at various times. All senior team members had undergone disability-specific training within their respective disciplines and were established mental health clinicians and supervisors in the field of developmental disabilities.

#### Psychiatric evaluations

All participants received a psychiatric evaluation that included: an interview with the patient (rapport-building, modified mental status exam, observations, and interactions); interview with parents and/or care providers (chief complaint, developmental, educational, psychiatric and medical histories, present illness, review of systems); and a review of collateral information derived from patients’ medical records and as needed from teachers, other physicians, or vocational/residential staff. Diagnoses using DSM-IV-TR [27] criteria were made by attending professionals and agreed upon by the clinical team. The few disagreements in diagnoses were discussed among team members at case conferences until consensus was achieved.

#### Chart review

Trainees in clinical psychology reviewed the participant’s records in order to informally verify symptoms associated with psychiatric diagnoses. This approach was used as the clinics did not administer symptom checklists. The trainees also noted if marked motoric slowing was present in speaking or performing everyday routines.

## Results

### Types and frequencies of psychiatric diagnoses

Table 2 presents the DSM-IV-TR diagnoses for adolescents and young adults in each group. As some diagnoses were relatively infrequent, diagnoses were classified into broader categories in order to conduct meaningful statistical analyses. When pertinent, however, Table 2 also notes specific diagnoses subsumed under these broader categories.

**Table 2:** **Number and percentages of primary psychiatric diagnoses in Down syndrome versus other ID groups**

	**Down syndrome (** ***N*** **= 49)**		**Intellectual disabilities (** ***N*** **= 70)**		
	***N***	**%**	**N**	**%**	***X*** ^**2**^ **(1),** ***p***
Psychosis NOS	17	35%	9	13%	6.46^**^
Depression with psychosis	4	8%	0		5.33^*^
Depressive disorders	7	15%	7	10%	.12
Bipolar disorder	2	4%	20	29%	14.34^***^
Anxiety disorders^a^	9	18%	7	10%	.20
Impulse control disorder^b^	10	20%	27	38%	4.59^*^

^*^ *p* < .05; ^**^ *p* < .01, ^***^ *p* < .001.

^a^Anxiety disorders included obsessive-compulsive disorder (DS = 4; ID = 2) with remaining diagnoses reflecting generalized anxiety disorder and one case of separation anxiety.

^b^Impulse control disorder included five participants with both ADHD and impulse control disorders (DS = 2, ID = 3). Depressive disorders consisted of major depressive episodes, with one case of dysthymic disorder in the ID group.

Chi-square analyses (2 × 2; group by yes/no psychiatric disorder) were conducted to compare diagnostic categories across groups. As shown in Table 2, those with Down syndrome versus other intellectual disabilities had significantly higher rates of psychosis NOS (35% versus 13%), with an additional 8% of those with Down syndrome having depression with psychotic features.

The following is a brief case vignette that exemplifies concerns seen in patients with Down syndrome, considering the relatively high rate of psychosis in this group. Psychosis was characterized in all cases by apparent auditory or visual hallucinations and/or delusions, withdrawal, and diminished communication. Other salient and more variable symptoms included restlessness, agitation, irritability, and impaired adaptive skills.

#### A case vignette exemplifying psychosis NOS in Down syndrome

J.M., a 22 year-old female with Down syndrome and mild intellectual disabilities (IQ = 58), was a recent high school graduate who had been a well-liked student. She lived with her mother, enjoyed caring for her dog and listening to music, and functioned independently in most aspects of personal grooming and daily living. After her graduation, she attended a series of job skills workshops, but she was not employed and was on a wait list to be evaluated for employment eligibility. After multiple months at home, her mother observed J. becoming increasingly irritable and withdrawn, culminating in a period of 4 days where she refused to change her clothes and slept with her light on. J. began talking to herself and ‘hearing voices inside my head’ and from inside the toaster. Her grooming deteriorated markedly, her sleep was disturbed, she became restless and agitated, and had prolonged conversations with imaginary others, including showing them her belongings. She reverted to sleeping with her mother and keeping all the lights on at night because she was afraid the voices would harm her. J. was hospitalized and responded well to antipsychotic medication, yet she was fearful that the voices would return. Her mother altered her work schedule to be more available during the day and hired her niece to be with J. in her absence. Her mother arranged for J. and her niece to volunteer a few hours a week at an animal shelter, though J. was not motivated to do so.

Compared to those with Down syndrome, the ID group had higher rates of bipolar (4% versus 29%) and impulse control disorders (20% versus 38%). Based on the chart review, both disorders were characterized by aggressive and destructive behavior, irritability, and angry outbursts. Those with bipolar disorder, however, also had depressed mood and bouts of restlessness, impatience, rapid speech, and making unrealistic demands.

The Down syndrome and ID groups had similar rates of depressive (15% versus 10%) and anxiety (18% versus 10%) disorders. Depression was characterized by sadness, withdrawal, irritability, lack of interest or disengagement in usual activities, and impaired adaptive skills. Those with anxiety disorders manifested excessive generalized worries or specific fears, and approximately 50% had repetitive, compulsive-like behaviors.

### Demographic correlates: age, race, IQ level, gender

To facilitate analyses of possible age effects, the sample was divided into two age groups: 45 adolescents aged 13 to 19 (*M* age = 17.22 years, SD = 2.13) and 77 young adults aged 20 to 30 (*M* = 24.47 years, SD = 3.23). Chi-square analyses (age group × psychiatric disorder) conducted within each group revealed that compared to adolescents, young adults with Down syndrome were less likely to have an impulse control disorder (35% versus 10%, respectively, *X*2 (1) = 5.20, *p* < .05). No other significant age effects were found.

**Figure 1:**
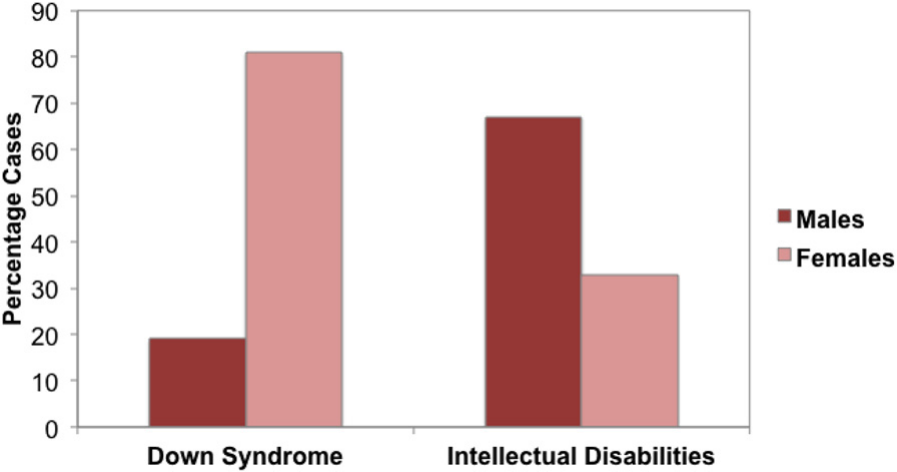
**Gender differences in psychosis (psychosis NOS plus depression with psychotic features) in Down syndrome versus ID groups.** The *X*-axis indicates groups by gender and the *Y*-axis the percentage of patients with psychosis-NOS diagnoses.

No significant associations were found between level of intellectual impairment or race/ethnicity for any psychiatric diagnosis in either the Down syndrome or ID groups. However, two gender effects emerged in the Down syndrome group. As shown in Figure 1, a full 81% of Down syndrome patients with psychotic symptoms were females; *X*2 (1) = 13.50, *p* < .001. In contrast, no significant gender differences in psychosis were seen in those with other IDs, *X*2 (1) = .005. Second, the majority (90%) of those with Down syndrome and impulse control disorder were males, *X*2 (1) = 7.92, *p* < .01, and although males comprised 65% of impulse control diagnoses in the other ID group, this was not significant, *X*2 (1) = .02.

### Marked slowing

Extreme slowing was seen in 17% of the Down syndrome group. These nine patients (four males, five females) had diagnoses of psychosis (*n* = 3), depressive (*n* = 4), or anxiety disorders (*n* = 2). They displayed excessive slowing in completing everyday routines (for example, taking hours to get dressed or eat) and long latencies (for example, 10 to 20 min) in responding to simple, previously mastered questions, even with repeated prompts.

## Discussion

This preliminary, descriptive study is the first to address a long-standing research gap on psychiatric disorders in adolescents and young adults with Down syndrome and other intellectual disabilities. Although not applicable to the population of those with ID or Down syndrome, the young patients in this study had high rates of such severe psychiatric conditions as psychosis, bipolar, mood, and impulse control disorders. Findings offer important insights into atypical aspects of the Down syndrome phenotype, with group and gender differences that encourage new avenues of future research.

Unexpectedly, psychosis NOS was seen in 35% of the 49 patients with Down syndrome, significantly higher than the 13% with psychosis in the ID group. An additional 8% of patients with Down syndrome had depression with psychotic features. Although the rate of psychosis in the ID group is consistent with previous clinical studies [28,29], psychosis is not generally reported in population-based or community samples of people with Down syndrome [18]. Psychotic symptoms have, however, been reported in hospitalized or clinic adult patients with Down syndrome and in older adults with dementia. For example, auditory hallucinations were reported in 45% of 22 adult patients with Down syndrome and depression [30], and Urv *et al.* [5] identified delusions or hallucinations in up to 79% of older adults over the age of 50 with possible or definite dementia. The present study, however, identified psychotic symptoms in a much younger cohort.

Two cautionary notes are in order about psychotic symptoms in people with Down syndrome or other IDs. First, some cognitive processes in people with intellectual impairments could easily be confused with delusions or hallucinations, but are instead consistent with mental age expectations and pre-operational levels of cognitive development [31]. These processes include a blurring of what is real versus pretend, having fantasies or imaginary friends, and engaging in overly concrete or magical thinking. Second, persons with Down syndrome often talk or mumble to themselves. Glenn and Cunningham [32] found that 91% of a sample of 77 young adults with Down syndrome either talked to themselves now or had done so in the past. Self-talk was often used to guide problem solving or revisit previous conversations and activities, and it was not related to social or behavioral problems. Both cognitive development and benign self-talk thus need to be taken into account when evaluating possible delusions or hallucinations in people with Down syndrome or ID.

Unexpectedly, females comprised a full 81% of Down syndrome patients with psychosis. Gender differences in psychosis are not found in the general population [27], nor were they seen in our ID group. As such, the preponderance of psychosis in young women with Down syndrome may simply be a function of chance. On the other hand, we examined the 22 cases of major depression in adults with Down syndrome reported by Meyers and Pueschel [15] and discovered that females comprised 70% of those with hallucinations.

Women with Down syndrome may be more susceptible to early aging, as well as premature menopause and reduced levels of estrogen [33,34]. Schupf *et al.* [35] found that women with Down syndrome and specific polymorphisms in the estrogen receptor gene 1 were three times more likely than women without these polymorphisms to develop Alzheimer’s disease. Although speculative, alterations in estrogen receptors and reduced estradiol availability may play a role in aberrant cognitive or emotional functioning in younger women with Down syndrome.

Relative to patients with ID, those with Down syndrome did not show higher rates of anxiety or depressive disorders. A well-controlled study of adults with Down syndrome who averaged 43 years of age also found rates of depression that were similar to others with ID [18]. However, high rates of depression or depressive symptomatology have previously been reported in older adults with Down syndrome [12-15], including withdrawal, irritability, mutism, and urinary incontinence [5,18]. These symptoms require careful evaluation as they may also signal the beginning stages of dementia. Advancing age may thus be a risk factor for depression in Down syndrome, yet the course of depressive symptoms from young to older adulthood is largely unknown.

Prominent slowing was observed in 17% of the Down syndrome group, and only in those with psychosis, anxiety, or depression. It was common for these individuals to take hours to complete otherwise routine grooming or feeding activities, to passively refuse to engage in activities they previously enjoyed, and to have pronounced delays (up to 20 min) in responding to simple queries that they had previously mastered (for example, ‘What is your name?’, ‘What color are your socks?’). Charlot and colleagues [23] attributed such ‘obsessional slowness’ in their patients to an extreme form of obsessive-compulsive disorder (OCD) and possible dopaminergic dysfunction.

However, none of the patients with marked slowing in our sample met criteria for OCD and instead bear a striking resemblance to two case reports of Down syndrome and catatonia reported by Jeb and Ghaziuddin [36]. These adolescent females had withdrawal, hallucinations, blunted affect, posturing, and psychomotor slowing, and they responded well to conventional treatment for catatonia. On an intriguing historical note, in 1946, Rollins [37] described ‘catatonic psychosis’ in 29% of 73 institutionalized adolescents and young adults with Down syndrome characterized by regression in speech, toileting and feeding skills, docility, inertia, apathy and withdrawal, motor rigidity, and unpredictable bouts of excitation. Although the diagnosis of catatonia may be underrecognized in contemporary psychiatric practice [38], it is much more common in those with versus without intellectual disabilities [39]. Future studies are needed that probe neurological, motor and physiological functioning in individuals with Down syndrome, and apparent catatonia.

Consistent with previous studies, impulse control disorders were more common in the ID versus Down syndrome group; these were the most frequently occurring diagnosis in our ID sample. Poor impulse control and aggressive behaviors in people with ID remain the leading reasons for caregivers to seek specialized care [40,41]. As in previous work, impulse control problems in our Down sample were more common in males and in adolescents versus young adults [15,19,20,30].

Relative to those with Down syndrome, the ID group had significantly higher rates of bipolar disorder (4% versus 29%, respectively). Relatively high rates of bipolar disorder have also been identified in other specialized psychiatric clinics for persons with ID, ranging from 26.5% to 42% of 200 and 166 patients with ID, respectively [41,42]. In contrast, studies conducted abroad find much lower rates of bipolar in ID samples, from 1% to 3% [28,43,44], raising the possibility that ID studies in the US mirror the increase in bipolar disorder diagnoses among children or youth in the general population. Debates continue about the extent to which chronic irritability, impulsivity, and explosiveness are valid indices of bipolar disorder in children or youth [45], or are instead subsumed under a new DSM-5 diagnosis ‘disruptive mood dysregulation disorder.’ Future research on bipolar diagnoses in youth with ID stands to benefit from these ongoing debates.

This study had several limitations. The sample size was relatively small, primarily because of our restricted age range and focus on patients in specialized psychiatric clinics. Second, clinicians did not utilize standardized psychiatric interviews. Even though clinicians were well trained in ID and also used a team approach, they may have overlooked concerns that are required probes in standardized interviews. Third, we were not able to obtain systematic data on the types or dosages of prescribed medications. Psychotropic medications have, however, been studied in much larger samples of people with ID and consistently show high levels of anticonvulsant and antipsychotic drug use relative to antidepressants or anxiolytics, as well as high rates of polypharmacy [29,46,47]. Future large-scale studies are needed that differentiate psychotropic medication use in specific etiologies, including Down syndrome.

The study did not formally measure relations between psychiatric symptoms and specific life events. Anecdotally, we noted that many young adults with Down syndrome had graduated from high school but were then left with little or nothing to do during the day. These informal observations warrant further study, as isolation and a lack of stimulating cognitive, physical, or recreational activities are risk factors for poorer outcomes in typical aging, as well as in depression and dementia [48,49]. A final limitation is that the clinics were not set up to conduct in-depth medical evaluations. Even so, clinicians ruled out medical conditions that are known to contribute to emotional or behavioral problems in the ID population, such as undetected or untreated pain, constipation, reflux, poor sleep, low thyroid, and untreated infections [50,51].

## Conclusions

Although clinic samples are not representative of broader populations, this study nonetheless highlights an urgent need for further research on psychiatric problems in youth and young adults with ID and Down syndrome. Work is especially needed on the high rates of apparent bipolar disorder in youth with ID, including the extent to which they respond to conventional bipolar treatment, have positive family histories of bipolar illness, or could instead be diagnosed with other disorders, including the new DSM-5 disruptive mood dysregulation disorder.

Future research is also needed on the pronounced withdrawal, psychosis, and apparent catatonia in some patients with Down syndrome. This research needs to identify the onset and course of such symptoms and their associations to aberrant neurologic, hormonal and neuroendocrine functioning, altered gene expression, acute or chronic illnesses, stressful life events, and inadequate environmental stimulation. From 1946 to today, different terms have been used to describe subsets of individuals with Down syndrome and severe clinical presentations, including catatonic psychosis, young adult disintegrative syndrome, depression, psychosis, catatonia, regression, and obsessional slowing. Although not well understood, research on these severe psychiatric presentations in youth and young adults stand to inform treatment options and add new dimensions to our phenotypic understandings of Down syndrome.
